# Lead Toxicity Risks in Gunshot Victims

**DOI:** 10.1371/journal.pone.0140220

**Published:** 2015-10-28

**Authors:** Gabriel Costa Serrão de Araújo, Natália Teixeira Mourão, Igor Natário Pinheiro, Analúcia Rampazzo Xavier, Vinicius Schott Gameiro

**Affiliations:** 1 Hospital Universitário Antônio Pedro, Faculdade de Medicina, Universidade Federal Fluminense, Niterói, RJ, Brazil; 2 Hospital Central da Polícia Militar, Rio de Janeiro, RJ, Brazil; Institute for Health & the Environment, UNITED STATES

## Abstract

**Background:**

Gunshot wounds require surgeons to decide whether to remove or leave bullet fragments in the body. Surgeons also decide how to follow up with patients who have lead fragments retained in their body. Current literature recommends to remove only intra-articular fragments without the need for a follow-up for patients with the metal retained. Therefore, this study investigates chronic lead toxicity for gunshot wounds.

**Methods:**

The study was performed in the metropolitan area of Rio de Janeiro/Brazil, between 2013 and 2015. It was a case-control study that included 45 victims of gunshot lesions with metallic fragments retained for more than 6 months. The 45 controls were matched for gender, age, and race. We compared the lead blood levels and frequency of symptoms.

**Results:**

The control group had average blood lead levels of 2.17 μg/dL (95% Confidence Interval [CI]; 1.71–2.63) and median 2.1 μg/dL. The case group had average values of 9.01 μg/dL (CI; 6.07–11.96) and median values of 6.5 μg/dL with p-values < = 0.001. The case group reported the following more frequently: irritancy, bad mood, headache, memory losses, daylight drowsiness, myalgia, weakness, abdominal pain, joint pain, trembling, tingling limbs. There was statistical significance for the differences of symptoms frequencies and for odds ratio between groups.

**Conclusions:**

Although the mean lead levels found were lower than the current laboratory references, low levels have been associated with both rising morbidity and mortality. The WHO stated: “There is no known level of lead exposure that is considered safe”. In conclusion, this work showed that bullets retained in the body are not innocuous. There are impacts in the blood lead levels and symptoms related to it, even with few fragments, extra-articular located or existing with low blood lead levels.

## Introduction

The high incidence of gunshot wounds has required surgeons to be skilled in treating the wide variety of lesions caused by bullets. Treatment requires the decision to remove or leave the metallic fragments retained in the body.[1] Sometimes, removal demands difficult surgical approaches, with extensive tissue dissection and high morbidity in a patient that is debilitated by the trauma.

When the metal stays retained in soft tissues, there is a foreign body reaction that forms a fibrous capsule that contains it and blocks the contact to blood and the lead (Pb) input. However, when the fragments are retained in a joint, the synovial fluid blocks the foreign body reaction, stimulates the metal dissolution, and facilitates absorption.[2, 3]

The literature has few case reports of patients that present clinical signs of lead poisoning caused by gunshot wounds. Blood lead levels are not routinely monitored, but the few studies that were proposed to investigate it showed increases in the blood lead levels.[4–7] The main issue is that lead chronic exposure may cause symptoms that are rarely associated with poisoning by doctors, such as psychological alterations, headache, cramps, or weakness.[8] There are clinical manifestations on a number of systems, including the hematological system[9] such as anemia, basophilic stippling, or porphyria; the digestive system such as anorexia, vomiting, constipation, abdominal pain, or cramps; the cardiovascular system such as arterial hypertension; and the neurological system such as peripheral neuropathy or encephalopathy;[10] which may lead to death.[11]

This work, thus, was designed as a case-control study to compare blood lead levels of gunshot victims to unexposed age, gender, and ethnicity matched controls.

## Materials and Methods

The study was performed in the metropolitan area of Rio de Janeiro, Brazil, between November 2013 and May 2015. Two hospitals were enrolled in the study: the Antônio Pedro University Hospital of Fluminense Federal University and the Central Hospital of Military Police of the Rio de Janeiro State. Two groups of volunteers were compared. We used a convenience sample of patients with records in the two participant institutions. The case group had volunteers that suffered gunshot wounds and had metal fragments in the limbs, spine, or pelvic ring. Inclusion criteria included that the projectiles must be retained for at least 6 months, and they must be more than 18 years of age and be lucid and able to answer the questionnaire. The control group was paired by gender, age, and ethnicity. It included patients attended at the Orthopedic Department, older than 18 years, who had previously given permission to collect preoperative blood exams for elective procedures not related to gunshot wounds. Exclusion criteria was the same for both groups: professions associated to lead exposure, for example, metallurgic workers, battery factory workers, and shooting instructors. However, no one met these conditions and has to be excluded.

All doctors of the Orthopedic Departments in both institutions were informed about the study. Even though they attend all different conditions in orthopedics, they were invited to refer all gunshot victims to the researchers. Volunteers were also enrolled after having been attended to in the emergency. In those cases, they were invited to return after 6 months when they matched the inclusion criteria.

The control group was matched and enrolled after their pairs in the cases. Every time one patient was interned in the orthopedic nursery to treat any condition or had pre-operative exams scheduled, they were checked to see if they match the criteria to be paired. Ethnicity was divided into the following categories: Asian, black, brown, Indian or white. The criteria for age was a 5-year difference between the case pair. All volunteers were informed of the research and consented. The research project was submitted to and approved by an independent ethical committee, respecting the institutions guidelines and the international agreements for scientific experiments with human tissues, including the Declaration of Helsinki (1964) and their following recommendations of Fortaleza/Brazil (2013). The Ethics Research Committee of the Fluminense Federal University gave their approval for the study. They authorized the informed consent procedure, which was written obtained. Detailed information is documented under the approval/registration number 20597413.6.0000.5243.

Blood samples were collected by upper limb venipuncture. Four milliliters were stored in a plastic, lead free, vacuum tube with heparin. Lead dosage in the whole blood was determined by spectrometry, Agilent 7700 ICP-MS system, in a laboratory certified by The College of American Pathologists and The International Certification Network (IQNET and FCAV).

The questionnaire was filled out by the researchers during anamnesis. It collected data about demographic information, clinical conditions, medicine usage, lead poisoning symptoms and professional history. We directly asked about 22 symptoms that are known to be associated with lead exposure. They included the following: abdominal pain, mental confusion, headache, loss of appetite, weakness, tingling limbs, decreased libido, eyesight losses, metallic taste, vomiting, irritancy, bad mood, myalgia, trembling, limbs sensitivity alterations, daylight drowsiness, trouble sleeping, constipation, seizure, memory losses, joints pain, unstable gait.

The case group was submitted to plain radiographic examination to confirm the metal fragments retained and to collect information about number of fragments and location. For this group, we also collected information about the gunshot trauma as well as the date and hospitalization history. The influence of fragment dispersion was analyzed and separated into 3 groups: volunteers with 1 fragment retained; those with 2 to 10 fragments; and those with more than 10 fragments. The fragments were counted by the researchers in the plain radiographs.

Descriptive statistics were used to evaluate the data. The inferential analysis for qualitative variables was investigated by chi-squared test. When the test was inconclusive, we alternatively used the Fisher’s exact test. Inferential analysis for quantitative variables, “lead levels”, and “time since trauma” was performed by non-parametric tests of Mann-Whitney to compare 2 groups and Kruskall-Wallis test for multiple groups comparison. The non-parametric tests were used because the Shapiro-Wilk test rejects the hypothesis of normal distribution for those samples. Both groups compared were treated as independent samples for statistical analysis. The matching procedure was used to homogenize the groups for confounding factors. Statistical significance was defined as p-value < 0.05. All data analysis was performed using SPSS software version 22.0.

## Results

**Fig 1:**
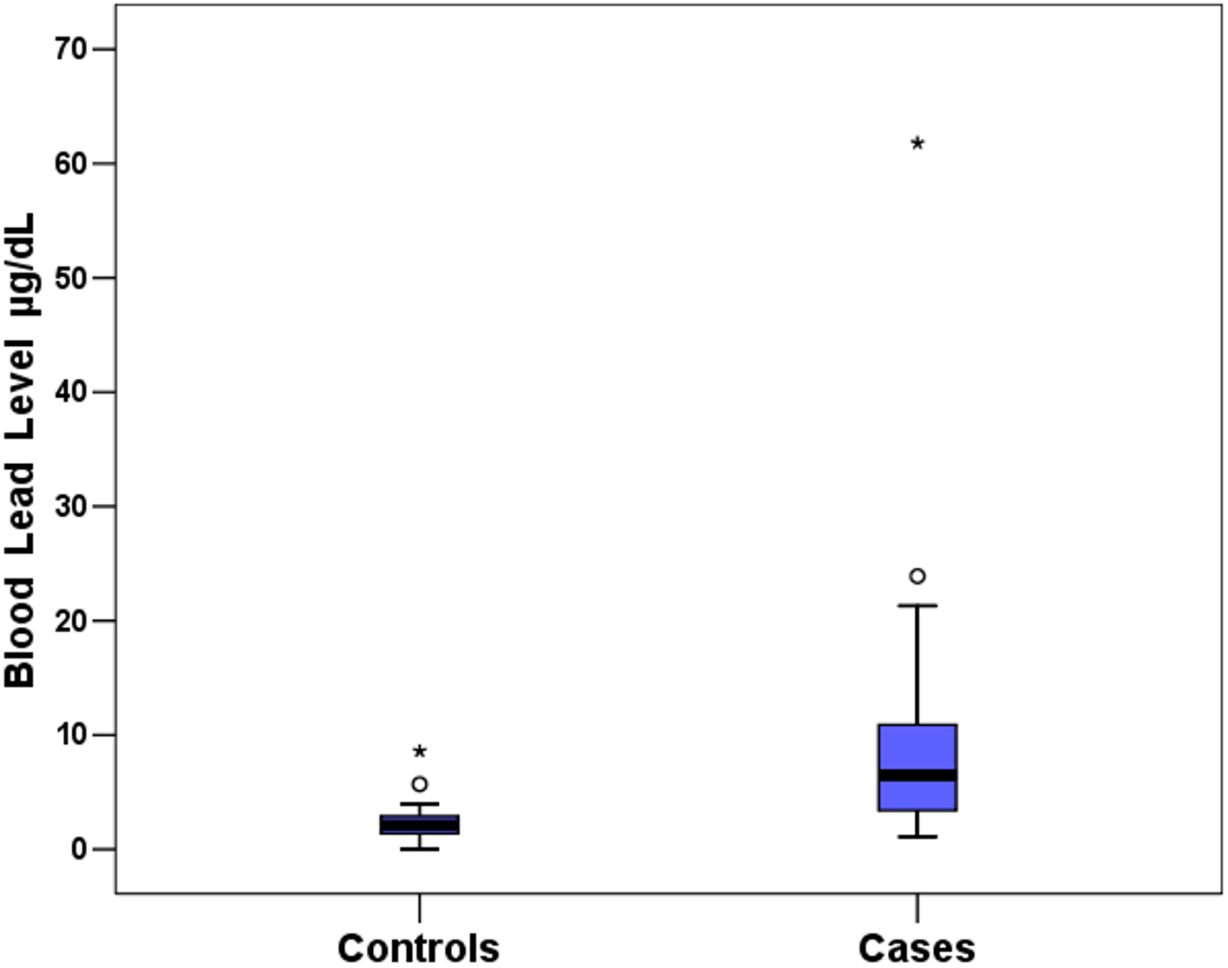
A box plot to represent the samples distribution for blood lead levels.

45 male subjects were enrolled in each group. There was no woman assessed as volunteer. The demographic distribution for ethnicity was the following: 5 black, 14 pardo (brown), and 26 white. The matching method resulted in mean age of 38 (range from 18 to 69) years in the case group and 39 (18–66) in controls. Statistical analysis for lead blood levels resulted in significant differences between case and control groups. The average blood lead levels were 2.17 μg/dL (95% CI; 1.71–2.63) with median 2.1 μg/dL for the control group, and the average levels were 9.01 μg/dL (CI; 6.07–11.96) with median 6.5 μg/dL for the cases with p value < 0.001. Fig 1 is a box plot for lead levels, where it is possible to see 2 outliers samples. The case group had a volunteer with 61.8 μg/dL who had a multi-fragmentary knee fracture with bullets retained intra-articular. The control outlier had a blood lead level of 8.6 μg/dL, but we did not found any biological explanation for it. We tested the mean differences without the discrepant samples and verified that groups were also significantly different (p < 0.001). Table 1 presents descriptive statistics with measures of centrality and dispersion.

**Table 1:** Descriptive statistics for blood lead levels (μg/dL).

Leads Level Statistics	Control Group	Case Group
Size of Group (n)	45 (50%)	45 (50%)
Mean	2.17	9.01
Confidence Interval for Mean (95% level)	(1.71; 2.63)	(6.07; 11.96)
Median	2.10	6.50
Standard Deviation (SD)	1.53	9.80
Mimimum	0.0	1.1
Maximum	8.6	61.8
Variation Coefficient (SD/Mean)	0.70	1.09
Significance from Shapiro Wilk Test	< 0.001	< 0.001
Significance from Mann-Whitney Test (comparing Control and Case Groups)	< 0.001	

The frequencies of symptoms were compared between the controls and the cases and for some symptoms, a significant difference was found. The strength of those associations are described in Table 2.

**Table 2:** Associations of symptoms and the presence of bullets retained.

Symptoms	Frequency (Control Group)	Frequency (Case Group)	p-value (Chi-Squared Test)	Odds Ratio (OR)	Confidence Interval for OR
**Memory Losses**	**1 (2.2%)**	**12 (26.7%)**	**0.001**	**16.0**	**(1.98; 129.27)**
**Irritancy**	**1 (2.2%)**	**10 (22.2%)**	**0.004**	**12.6**	**(1.54; 102.97)**
**Weakness**	**1 (2.2%)**	**10 (22.2%)**	**0.004**	**12.6**	**(1.54; 102.97)**
**Trembling**	**1 (2.2%)**	**10 (22.2%)**	**0.004**	**12.6**	**(1.54; 102.97)**
**Tingling limbs**	**1 (2.2%)**	**10 (22.2%)**	**0.004**	**12.6**	**(1.54; 102.97)**
**Bad Mood**	**3 (6.7%)**	**19 (42.2%)**	< **0.001**	**10.2**	**(2.75; 38.00)**
**Joints Pain**	**1 (2.2%)**	**7 (15.6%)**	**0.029** *	**8.1**	**(1.05; 68.86)**
Metallic Taste	1 (2.2%)	5 (11.1%)	0.203	5.5	(0.62; 49.18)
**Headache**	**6 (13.3%)**	**19 (42.2%)**	**0.002**	**4.7**	**(1.67; 13.48)**
**Myalgia**	**4 (8.9%)**	**14 (31.1%)**	**0.008**	**4.6**	**(1.39; 15.45)**
**Daylight Drowsiness**	**6 (13.3%)**	**14 (31.1%)**	**0.043**	**2.9**	**(1.01; 8.53)**
Eyesight Losses	5 (11.1%)	8 (17.8%)	0.368	1.73	(0.52; 5.76)
Loss of Apetite	2 (4.4%)	3 (6.7%)	1.000	1.54	(0.24; 9.66)
Trouble Sleeping	10 (22.2%)	12 (26.7%)	0.624	1.27	(0.48; 3.34)
Constipation	4 (8.9%)	3 (6.7%)	1.000*	0.73	(0.15; 3.48)
**Abdominal Pain**	**0 (0.0%)**	**7 (15.6%)**	**0.012** *	**-**	**-**
Vomiting	1 (2.2%)	0 (0.0%)	1.000*	-	-
Seizure	0 (0.0%)	1 (2.2%)	1.000*	-	-
Mental Confusion	0 (0.0%)	3 (6.7%)	0.242*	-	-
Unstable Gait	0 (0.0%)	5 (11.1%)	0.056*	-	-
Limbs Sensitivity Alterations	0 (0.0%)	5 (11.1%)	0.056*	-	-
Decreased Libido	1 (2.2%)	0 (0.0%)	1.000*	-	-

* **Fisher exact test.**

We found no association between the number of symptoms in each volunteer and the time that the bullets were retained. The Spearman correlation between the variables was 0.209 (p-value = 0.167). We also searched for an association between the number of symptoms in each volunteer and the lead levels; the Spearman correlation was weak (0.26) but significant (p = 0.012).

The analysis for correlation between lead levels and time of bullet retention resulted in a Spearman correlation of -0.38 (p = 0.01). Therefore, blood lead levels are weakly correlated to bullet retention time.

**Fig 2:**
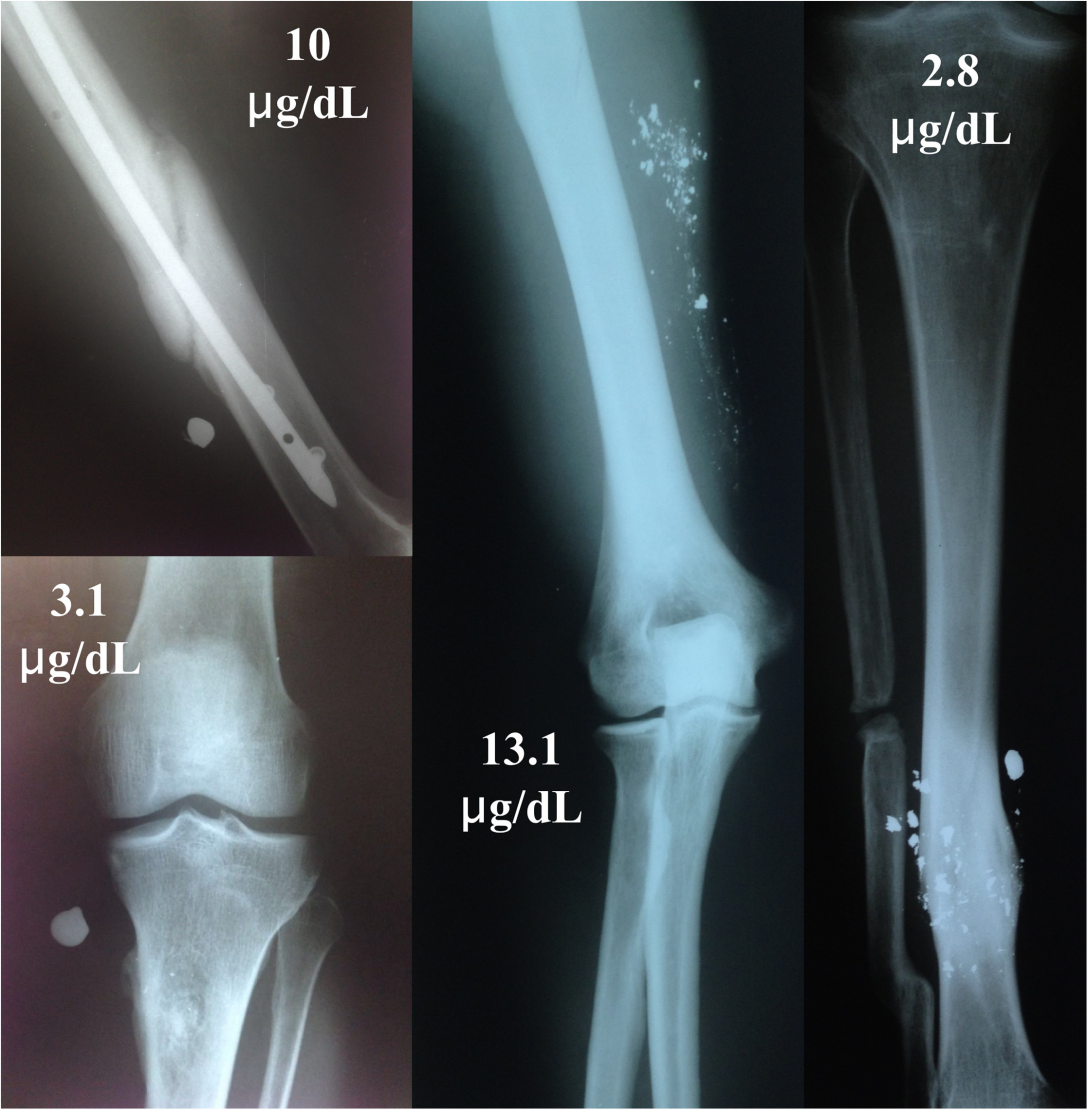
Examples of volunteers with different number of bullets fragments retained and their respective blood lead levels.

The dispersion pattern effect of retained bullet fragments was analyzed using the Kruskall-Wallis test to compare the lead levels between 3 groups: volunteers with 1 fragment retained (12 cases); with 2 to 10 fragments (24 cases); and with more than 10 fragments (9 cases). The mean lead levels from subgroup of volunteers with 1 fragment retained was 9.2 (± 16.7) μg/dL with median 3.55 μg/dL; for the subgroup of volunteers with 2 to 10 fragments, the mean level was 8.8 (± 6.2) μg/dL with median 7.6 μg/dL; and from subgroup of volunteers with more than 10 fragments, the mean lead level was 9.3 (± 5.9) μg/dL with median 7.4 μg/dL. Fig 2 shows examples of the different number of fragments retained and their respective lead levels. The statistical test resulted in a p-value of 0.233, which suggests that the lead level is not associated to the number of bullet fragments in the body.

While investigating the sites of the retained bullets, we found that the fragments were often widely distributed in multiple tissues (*i*.*e*., muscles, bones, subcutaneous, joints and spinal canal). No relations were found between lead levels and the sites of the retained bullets. A larger sample would be necessary for this analysis.

## Discussion

The industrial application of lead has impacted the blood levels of the general population. Surveys in the USA reported that in the 1960s blood levels were higher than 20 μg/dL and lowered to 13 μg/dL in that late 1970s until reaching 3.5 μg/dL in the last decade.[12] A Brazilian population study published this year[13] reported an average of 1.97 μg/dL (95% CI; 1.9–2.04 μg/dL). Both studies also identified gender, race and age as risk factors for blood lead levels. In agreement with the literature, our control group presented a mean level of 2.17 (Standard Deviation; ± 1.53) μg/dL.

The first study that was designed to describe the association between gunshot wounds and blood lead levels, was performed by Farrell and col[5] in 1999. They compared two groups of 15 patients in a matched case-control study. They found a mean (± SD) of 17 (± 9.78) μg/dL for the cases and 7 (± 3.77) μg/dL for the controls; these were found to be statistically significantly different. Both means were higher than in our study, indicating a higher environmental exposure. It is important to highlight that: their data was collected during the 90’s, the location of the study was not given, and the professions of half of the volunteers were not recorded. These factors could contribute to the differences in our mean values.

McQuirter et al.[6] prospectively studied a volunteer sample of 451 subjects with retained bullets. There was a tendency of blood lead levels' elevation during the first 6 months after the trauma and stabilization after that time. For that reason, in our transversal study we included only volunteers with more than 6 months after trauma. They considered that three factors were as predictive to elevated blood levels: number of fragments, torso bone fracture and bullet fragments in the humerus. In our study, neither the number of bullet fragments nor their location were correlated to intoxication. They recommended, and we agree, that patients should have the lead blood levels recorded at hospital admission and be advised to obtain yearly blood lead determinations thereafter. Despite the methodological differences between the prospective/transversal studies, they found a prevalence at 12 months after trauma of 20,1% of individuals with blood lead levels above 10 μg/dL and 2,6% for those above 20 μg/dL. We found, respectively, 31,11% and 6,66%. Our controls did not values above those limits.

A case-control study[7] with 120 patients with extra-articular retained missiles reported blood lead mean values (95% CI) of 6.71 (5.68–7.74) μg/dL and 3.16 (2.79–3.53) μg/dL in case and control groups, respectively, with significant differences verified using a matched pairs t-test (p = 0.0001). They also demonstrated an association between recent fractures and elevated lead levels. The authors suggested that the periodically blood lead levels screening may benefit those patients, especially after episodes of increased metabolic stress. This work was the most similar to our study, including methods and results. They also did not find relationships between the number of fragments and the blood lead elevation. However, in contrast to our study, they did not verify more symptoms in their cases.

In 2014, Moazeni et al.[14] reported a comparison of 25 patients with some retained lead pellets in their bodies and the same number of volunteers without similar lead exposure. The results showed lead levels of 29 (± 12.8) μg/dL and 25.3 (± 6.4) μg/dL in case and control groups, respectively, without any significant difference (p = 0.3). But, they confirmed the previous studies that found that there is no association between long time retention of bullet fragments in the body and rising lead levels. Although, they found that patients who had more lead pellets in their bodies had higher blood lead levels (p = 0.025). Our study found whole blood lead levels in the case group to be between 1.1 μg/dL and 61.8 μg/dL, with mean and standard deviation of 9.01 ±9.8 μg/dL and median 6.5 μg/dL. The control group mean was 2.1 (±1.53) μg/dL with median 2.1 μg/dL. The statistical analysis showed significance differences in the lead levels between the two groups (p < 0.001). These differences suggest that their controls were more exposed to lead than ours controls. Their discrepant results could perhaps be explained by an environmental or occupational exposure that was not well controlled.

Fig 2 shows examples of individuals with higher blood lead levels with only one fragment retained and another with multiple, widely dispersed fragments. The figure also shows the opposite situation where there is no correlation between the number of fragments and the blood lead concentration. These findings are consistent with Nguyen *et al*.[7], who compared patients with retained bullets until 51 years (mean of 4.6 years). The reports of Moazeni *et al*.[14] and McQuirter *et al*.[6] found relations between exposure time and blood lead levels. They both evaluated patients with less than 6 years since trauma. This apparent divergence in the literature can be explained by pathophysiology. Initially, the metal retained induce the inflammatory process, the foreign body reaction and it is transported to the bone stocks. After that, there is a stabilization between the absorption rates, blood distribution, renal elimination, bone storage and stocks mobilization. That is why, theoretically, after long periods, the dispersion of fragments do not influence the blood lead levels.

The Brazilian regulatory standard number 7,[15] which was published by the Ministry of Work and Employment, is the official laboratory reference for lead exposure. It established 40 μg/dL as the accepted lead blood level for unexposed individuals. This guideline considers 60 μg/dL as the maximum allowed biological index for workers exposure. Although cases presented mean levels were lower than the laboratory references, low levels have been associated with rising both morbidity and mortality. Along this line, the World Health Organization stated: “There is no known level of lead exposure that is considered safe”.

Sanders et al.[16] and Florea at al.[17] published wide reviews of the literature regarding the neurotoxic effects of lead exposure. This metal can exist throughout almost every organ and system. The toxicity in target organs is widely variable but is especially important in the central nervous system. Lead (Pb2+) can mimic calcium (Ca2+) functions and modifies their pre- and post-synaptic effects. There are concerns about the toxic impact on cognition and mortality in chronic exposures. Coon et al.[18] studied chronic Pb exposure and confirmed that it is a risk factor for Parkinson’s disease. Khalil et al.[19] followed 533 women aged 65–87 years in a 12-years prospective cohort study. They compared individuals with blood lead concentrations above and below 8 μg/dL. A three-fold risk in coronary heart disease mortality and 73% higher risk for total mortality risk were found. The odds ratio of diastolic hypertension was 8.1 comparing women with blood lead levels of 4.0–31.1 μg/dL to lower ones 0.5–1.6 μg/dL. A prospective cohort study[20] found that men with one standard deviation increase in blood lead level were associated with a 1.27 (CI; 1.01–1.59)-fold greater risk for ischemic heart disease. Fang et al[21], in their case-control study, found an increased risk for amyotrophic lateral sclerosis, even in extremely low blood lead levels. Their means levels were 1.76 μg/dL (range, 0.32–6.90) among controls and 2.41 μg/dL (range, 0.72–7.58) among cases. An editorial[22] at the periodical Circulation alerted: “Low-level environmental exposure to lead unmasked as silent killer”. It discussed that levels below 10 μg/dL can lead to a higher risk of death. They also observed that levels as low as 2.07 μg/dL, in adolescents, is a public health hazard.

The U.S. Centers for Disease Control and Prevention considers 10 μg/dL as the limit for children exposure, but Gilbert and Weiss[23] published their perspective to lower the limit to 2 μg/dL. The developing brain is very sensitive to lead toxicity. There are several studies that relate lower blood lead levels with a rising risk of poor reading ability, arithmetic and others academic performances.[24] Concerns about lead exposure during pregnancy and reduced intellectual development in children with prenatal lead exposure have been reported.[25] Although we did not have any women in our study, the feminine presence in the security forces is increasing worldwide. Women soldiers in the battle fronts are an especially susceptible population, because bullets retained can theoretically jeopardize their children. Another reason for this concern is that lead in the skeleton is mobilized during pregnancy, as demonstrated by Gulson an cols.[26] The blood lead elevation is expected during pregnancy and is also a potentially harmful factor for the fetus.

Needleman et al.[27] reported a study comparing 194 youths aged 12–18 who were arrested and adjudicated delinquents to 146 non delinquent controls from high schools. They found significantly higher bone lead levels in the case group. Our study was not designed to compare violence behavior, but our case group reported the following more frequently: irritancy, bad mood, headache, memory losses, daylight drowsiness, myalgia, weakness, abdominal pain, joint pain, trembling, and tingling limbs. These symptoms can influence the social life and the quality of life of these individuals.

The main limitation of this study is inherent to all case-controls. We would like to follow-up a coorte with all patients received at the emergency since they arrived at the day of trauma. It would allow us to establish causative relations. However, in our region, gunshot victims are difficult to follow up with because usually they have security issues such as having their identity revealed. The case group included individuals who voluntarily participated. For that reason, it was not possible to evaluate gunshot victims who did not have personal concerns about their own health. Socioeconomic status is a common concern for studies on lead poisoning. Although, we work in a public hospital that offers free health assistance and, consequently, we attend to mainly the low-income population.

In conclusion, this work showed that bullets retained in the body are not innocuous. There are impacts in the blood lead levels and symptoms related to it, even with few fragments, extra-articular located or existing with low blood lead levels. These findings contribute to a new perspective on treating patients as well as recommendations to test blood lead and lead toxicity investigations. We also think that it is important to keep in mind the possibility to remove fragments. Nowadays, while the benefits and risks involved in removing fragments are not clear, the patients should be aware of the issues and take part in these decisions.

## Supporting Information

S1 Table Raw data table file with all information used for statistical analysis. (XLSX)
